# Seroprevalence of Pandemic Influenza H1N1 in Ontario from January 2009–May 2010

**DOI:** 10.1371/journal.pone.0026427

**Published:** 2011-11-14

**Authors:** Camille Achonu, Laura Rosella, Jonathan B. Gubbay, Shelley Deeks, Anu Rebbapragada, Tony Mazzulli, Don Willison, Julie Foisy, Allison McGeer, Ian Johnson, Marie LaFreniere, Caitlin Johnson, Jacqueline Willmore, Carmen Yue, Natasha S. Crowcroft

**Affiliations:** 1 Department of Surveillance and Epidemiology, Public Health Ontario, Toronto, Ontario, Canada; 2 Public Health Laboratories, Public Health Ontario, Toronto, Ontario, Canada; 3 Ottawa Public Health, Nepean, Ontario, Canada; 4 Toronto Public Health, Toronto, Ontario, Canada; 5 Dalla Lana School of Public Health, University of Toronto, Toronto, Ontario, Canada; 6 Department of Laboratory Medicine and Pathobiology, University of Toronto, Toronto, Ontario, Canada; 7 Department of Microbiology, Mount Sinai Hospital, Toronto, Ontario, Canada; University of Hong Kong, Hong Kong

## Abstract

**Background:**

We designed a seroprevalence study using multiple testing assays and population sources to estimate the community seroprevalence of pH1N1/09 and risk factors for infection before the outbreak was recognized and throughout the pandemic to the end of 2009/10 influenza season.

**Methods:**

Residual serum specimens from five time points (between 01/2009 and 05/2010) and samples from two time points from a prospectively recruited cohort were included. The distribution of risk factors was explored in multivariate adjusted analyses using logistic regression among the cohort. Antibody levels were measured by hemagglutination inhibition (HAI) and microneutralization (MN) assays.

**Results:**

Residual sera from 3375 patients and 1024 prospectively recruited cohort participants were analyzed. Pre-pandemic seroprevalence ranged from 2%–12% across age groups. Overall seropositivity ranged from 10%–19% post-first wave and 32%–41% by the end of the 2009/10 influenza season. Seroprevalence and risk factors differed between MN and HAI assays, particularly in older age groups and between waves. Following the H1N1 vaccination program, higher GMT were noted among vaccinated individuals. Overall, 20–30% of the population was estimated to be infected.

**Conclusions:**

Combining population sources of sera across five time points with prospectively collected epidemiological information yielded a complete description of the evolution of pH1N1 infection.

## Introduction

In Canada, the first cases of pandemic H1N1 2009 (pH1N1/09) were reported on April 26, 2009; two days later the first cases were reported in the province of Ontario [1], the largest province in the country. The number of reported cases in Ontario increased rapidly, with the peak of the first wave occurring by mid–May then tapering off in the summer months. The second wave began in the fall of 2009 and peaked during the last week of October. Starting October 26, 2009, Ontario began a mass pH1N1/09 vaccination program initially focusing on priority groups and expanding to the general population by November 16, 2009 [2]. By the end of January 2010, 8791 lab confirmed cases and 1843 hospitalizations associated with pH1N1/09 had been reported in Ontario [3].

Surveillance data based on laboratory confirmed cases capture only a fraction of the true cases of influenza since not all infected individuals are symptomatic, seek medical attention and provide specimens for laboratory testing. The extent to which surveillance reflects the true burden of disease was also affected by changes in the laboratory testing recommendations. Given the limitations in these data we designed a seroprevalence study with the following objectives: to estimate the community seroprevalence of pH1N1/09 in January 2009 before the outbreak was formally recognized; to assess the extent of community transmission of pH1N1/09 at multiple time points from January 2009 to the end of influenza season in April/May 2010; to identify the risk factors for infection with pH1N1/09, and; to assess the antibody response in individuals that were vaccinated during the second wave. Our aim was to develop an as complete as possible picture of the evolution of seroprevalence over the whole course of the 2009 H1N1 pandemic in Ontario.

## Methods

### Ethics Statement

The research protocol entitled “A Seroprevalence study of novel swine influenza A H1N1 among Ontarians” (protocol reference #24130) was granted approval by the Health Sciences Research Ethics Board at the University of Toronto, Canada. Written informed consent was obtained from participants.

### Study Populations

We obtained specimens from two sources and three populations at multiple time periods (table 1). Firstly, we recruited a prospective cohort of Ontario residents and followed them up after the first and second wave of pH1N1/09. Secondly, we assembled a repository of residual serum specimens submitted to Public Health Ontario Laboratories (PHOL) for preventable disease and prenatal screening at five time points from January 2009 to April/May in 2010.

**Table 1:** Summary of sample sizes time points for each of the study cohorts, January 2009 to May 2010.

	Jan-09	Feb-09	Mar-09	Apr–May-09	Jun-09	Jul-09	Aug–Sep-09	Oct-09	Nov-09	Dec-09	Jan-10	Feb-10	Mar-10	Apr–May-10
Timing of pH1N1 activity				- First cases in Ontario (04/28)- Peak of 1^st^ wave (mid-May)				- Peak of 2^nd^ wave (end Oct)- Priority groups pH1N1 vaccination program (10/26)	- General populationpH1N1 vaccination program (11/16)		-Significant decline in pH1N1 activity		1.1% pH1N1 positive of all respiratory specimens week of April 5^th^ [34]	
**Study Population**														
Cohort Study							1024							373***
Preventable disease screening*	383			383			505				838			790
Prenatal screening**				105			120					136		115

*Specimens obtained from Toronto, Kingston, Windsor and Hamilton public health laboratories.

**Specimens obtained from Toronto public health laboratories only.

***Only sero-negatives from wave 1 were invited to participate in the final phase.

### Prospective Cohort Study

We invited Ontario residents who were 18 years or older, available for follow–up in August/September 2009, able to communicate in English and answer an online questionnaire to participate in the study. Participants provided informed consent and completed a web-based questionnaire on health behaviours, health history and demographic information. Blood specimens were collected in serum separator tubes (SST tubes) at medical laboratory locations throughout Ontario. We aimed to recruit 1800 participants (600 from each of 3 age groups: 18–29, 30–64 and 65 years of age and older) in order to have 80% power to detect a difference in seroprevalence of 10%. We recruited through news releases, news articles, newspaper advertisements, emails to stakeholders, Google ad words and Facebook. Newspaper readership in the selected newspapers was over 1 million individuals per day, and at the time, the term “H1N1 flu virus” demonstrated a high percentage of all Google searches. In April 2010, study participants who were seronegative by hemagglutination inhibition assay (HAI) assay after wave 1 were invited to provide a second blood sample and complete another online questionnaire on risk factors, health status and vaccination history.

### Residual Specimens

In Ontario, all preventable disease and prenatal screening tests are performed by OAHPP laboratories. These specimens are submitted for a variety of reasons including occupational screening, requiring proof of immunity for school purposes, and screening of new immigrants. Prenatal screening is recommended for all pregnant women in the province. Residual sera held by OAHPP were randomly selected and had complete information on sex, age, and residence (appendix S1).

### Laboratory Testing

Sera were extracted from blood specimens and tested by HAI to determine antibody titres against the pH1N1/09 influenza strain (A/California/07/2009–like) and the 2008–2009 seasonal H1N1 influenza strain A/Brisbane/59/07 (Brisbane H1N1) to identify potential cross-reactivity. The HAI and microneutralization (MN) protocols were adapted from previously published World Health Organization (WHO) methods [4]. Briefly, the HAI assay was performed with 0.7% guinea pig erythrocytes and 4 HA units of virus. For MN, a twofold serial dilution was completed on the sera starting with 1∶40 dilution. Diluted sera and 100TCID50 infectious units of virus were added in equal amounts to each well in a U-shaped microtitre plate. The plates were incubated for two hours at 37°C to allow for virus- antibody interaction. Flat bottomed microtitre plates containing confluent monolayer Madin-Darby Canine Kidney (MDCK) cells (Diagnotic Hybrids Inc., Ohio, USA) were washed with virus growth medium. The virus-antibody mixture was added to the corresponding wells in the microtitre plate containing MDCK cell monolayer and incubated for a further 2 hours at 37°C. The contents were then removed and replaced with virus growth medium. The plate was then again incubated at 37°C and monitored for the appearance of cytopathic effects on days 3, 4, and 5. The reciprocal of the highest dilution of the antibody that inhibited the development of viral CPE was designated as the titre. Screening of the samples was done in triplicate wells and the titration was done in duplicate wells. Samples with a titre of 1∶40 or greater were considered seropositive for both assays [5]–[9].

### Statistical analysis

We calculated the proportion of seropositive participants for each age group, time period and study population with 95% confidence intervals according to the binomial distribution.

To assess the strength of the association between risk factors and positive seroprevalence status in the cohort study, we calculated unadjusted and age-adjusted odds ratios (OR) with 95% confidence intervals using logistic regression analysis. Multivariate logistic regression was fit to determine independent predictors and variable selection done was completed using the Hosmer-Lemeshow forward model building strategy. Briefly, univariate logistic regression for each independent variable was conducted. Variables that were significant using a cut-off of 0.2 were considered as candidates in the multivariate model. Variables were added based on level of importance using magnitude of the odds ratio and a priori variables (age and sex). Variables were retained in the final multivariate model if they were significant (P<0.05) or determined a priori. Geometric mean titres (GMTs) were calculated for cohort study participants who provided samples post-wave 1 and at the end of the 2009/10 influenza season. For titres lower than 10 (<1∶10), GMTs were estimated by assigning a value of 5. Sensitivity and specificity of the HAI was calculated using the MN assay as gold standard. All analyses were completed in SAS version 9.1.

## Results

We collected residual serum specimens from 3375 individuals who had submitted during our time periods of interest. For the cohort study, 1486 people registered to take part in the study and of those, 53 (3.6%) withdrew primarily due to difficulties scheduling appointments to provide a blood specimen. Among the remainder, 1245 (86.9%) completed the online questionnaire and 1069 (74.6%) had a blood specimen collected. Forty-five participants who provided blood specimens after October 5, 2009 were excluded, leaving 1024 or 68.9% for analysis post wave 1. In April 2010, 941 seronegative individuals who were asked to provide another specimen. Thirty-eight (4.0%) actively withdrew and 518 (57.4%) did not respond leaving 385 in the cohort study for the post-wave 2 analysis (appendix S2).

Compared with the general population, preventable disease residual specimens were more likely to be female and living in the Toronto region (table 2). Cohort study participants were more likely to be female, white, born in Canada, university educated, a health care worker and older than 65 years of age (table 2).

**Table 2:** Demographic characteristics of each study population compared to the general population of Ontario.

Category	Number and Proportion of Participants n (%)						Proportion of Ontario's Population, % [35]–[37]
	Preventable Disease Residual Specimens (N = 2899)		Prenatal Residual Specimens (N = 476)		Cohort Study (N = 1024)		
<18*	494	(17)	93	(20)	-		24
18–29 years†	480	(17)	127	(27)	160	(17)	14
30–64 years‡	1387	(48)	256	(54)	525	(56)	49
65+ years	538	(19)	–		259	(27)	13
Female	2010	(69)	476	(100)	551	(58)	51
Male	887	(31)	0	(0)	392	(42)	49
Toronto	1128	(39)	249	(52)	232	(23)	21
Central East	820	(28)	154	(32)	227	(23)	29
Eastern	202	(7)	2	(<1)	207	(21)	13
Central West	486	(17)	55	(12)	175	(18)	19
South West	207	(7)	1	(<1)	115	(12)	12
North East	40	(1)	9	(2)	34	(3)	4
North West	10	(<1)	4	(1)	7	(1)	2
White	–		–		875	(93)	77
Non-white	–		–		70	(7)	23
University degree	–		–		553	(59)	20
No university degree	–		–		392	(41)	80
Health-care worker	–		–		138	(15)	5
Teacher (pre-school to grade 12)	–		–		42	(4)	4
Child care worker	–		–		5	(<1)	1
Other	–		–		750	(80)	90

*For prenatal residual specimens, only ages 10 to 17 years are included. For the Ontario population, includes those <20 years of age.

† For the Ontario population, includes those 20–29 years of age.

‡ For prenatal residual specimens, only ages 30 to 49 years are included.

For each study population, pH1N1/09 seroprevalence levels increased until after the second wave with the largest increases occurring in the younger age groups. Overall pre-existing seropositive levels ranged from 2.0 to 12.0%, with those 65 years and older having the highest levels. Following wave 1, seroprevalence in study populations ranged from 10.9 to 18.3% overall with the highest proportions seen in the prenatal residual samples and generally among those in younger age groups. At the end of the 2009/10 influenza season, overall seroprevalence levels ranged from 32.2% to 40.9%, but varied greatly between age groups. In the residual samples seropositivity dropped slightly between the end of the second wave and the end of the influenza season (January to May 2010) (table 3).

**Table 3:** Proportion seropositive by HAI assay by age group, time period and study population.

Study population	Time period	% Seropositive (95%CI)				
		<18 yrs	18–29 yrs	30–64 yrs	65+ yrs	Total
Cohort Study	3. Post-wave 1 pandemic		14.4 (8.9–19.8)	10.7 (8.0–13.3)	8.9 (5.4–12.3)	10.9 (9.0–12.8)
	5. End of flu season (only seronegatives from period 3)		45.2 (30.2–60.3)	43.7 (36.6–50.7)	33.3 (25.3–41.4)	40.0 (35.1–44.9)

		<18 yrs	18–29 yrs	30–64 yrs	65+ yrs	Total
Preventable disease residual samples	1. Pre-pandemic	2.0 (0.0–5.8)	7.4 (0.4–14.4)	4.1 (1.3–6.9)	12.0 (5.0–19.1)	6.0 (3.6–8.4)
	2. Early Wave 1 pandemic	3.7 (0.0–8.7)	7.4 (0.4–14.4)	8.1 (4.3–11.9)	15.6 (7.5–23.7)	8.9 (6.0–11.7)
	3. Post-wave 1 pandemic	21.6 (13.0–30.2)	12.5 (5.6–19.4)	11.0 (7.1–14.9)	19.0 (10.7–27.4)	14.5 (11.4–17.5)
	4. Post-wave 2 pandemic	63.8 (56.3–71.2)	38.8 (30.7–47.0)	36.8 (32.0–41.6)	35.9 (28.4–43.4)	42.1 (38.8–45.5)
	5. End of flu season	61.7 (53.7–69.7)	37.9 (30.0–45.8)	36.3 (31.4–41.3)	34.8 (26.8–42.7)	40.9 (37.5–44.3)

		<18 yrs	18–29 yrs	30–64 yrs	65+ yrs	Total
	1. Pre-pandemic	n/a	n/a	n/a	n/a	n/a
Prenatal residual samples	2. Early wave 1 pandemic	4.2 (0.0–12.2)	11.1 (0.0–23.0)	9.3 (1.5–17.0)	n/a	8.6 (3.2–13.9)
	3. Post-wave 1 pandemic	27.6 (11.3–43.9)	10.0 (0.0–20.7)	18.0 (8.4–27.7)	n/a	18.3 (11.4–25.3)
	4. Post-wave 2 pandemic	29.4 (14.1–44.7)	48.5 (31.4–65.5)	43.5 (31.6–56.0)	n/a	41.2 (32.9–49.4)
	5. End of flu season	83.3 (35.9–99.6)	32.4 (17.3–47.5)	27.8 (17.4–38.1)	n/a	32.2 (23.6–40.7)

**Figure 1:**
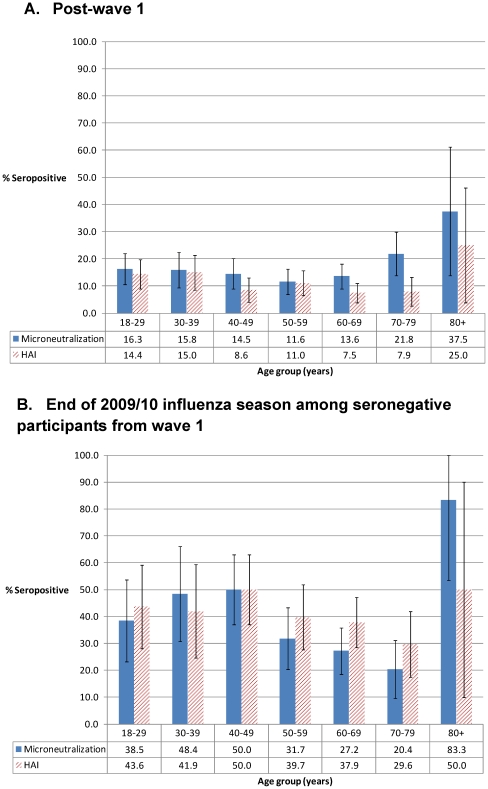
pH1N1/09 seroprevalence levels stratified by age group and serological assay among cohort study participants post-wave 1 (A) and among seronegative cohort study participants followed at the end of 2009/10 influenza season (B).

The MN assay indentified a larger number of infections compared with HAI, particularly among those 65 years of age and older (P<0.0001) (figure 1). The sensitivity of the HAI assay compared to the MN assay as a gold standard increased significantly from 53% (44%–60%) post-wave 1 to 82% (74%–88%) at the end of the influenza season (data not shown). In the cohort study, individuals 80 years and older had the highest pH1N1/09 seroprevalence following wave 1 and at the end of the 2009/10 influenza season as measured by HAI; however, confidence intervals were wide given the limited sample size within this age strata.

Table 4 shows the age-adjusted OR among the cohort study participants associated with each risk factor and pH1N1/09 seroprevalence following the first wave as measured by both HAI and MN assays. Independent predictors of serological status by HAI were: being a Toronto resident, experiencing fever and cough since April 1^st^ 2009, receiving the 2008/09 influenza vaccine, and attending a large family gathering. Seropositivity by MN was significantly associated with reporting ‘flu-like symptoms’ or fever and cough and receiving the 2008/09 influenza vaccine. The OR for age showed younger individuals were less likely to be positive by MN status but more likely to be positive using the HAI; however, the confidence intervals overlapped. In the multivariate analysis receiving the 2008–9 vaccine was only significant using HAI (OR = 1.68 95% C.I 1.04–2.70) and did not reach significance using MN (OR = 1.35 95% C.I 0.91–2.02). Associations with large family gatherings were significant in the multivariate HAI analyses; however, no significant effects were found with travel or hand hygiene.

**Table 4:** Analysis of risk factors associated with pH1N1/09 seroprevalence among cohort study participants post wave 1 in August–September 2009.

Risk factor	HAI assay		Microneutralization assay	
	Age-adjusted OR (95% CI)	Multivariate* adjusted OR (95% CI)	Age-adjusted OR (95% CI)	Multivariate* adjusted OR (95% CI)
Male	0.91 (0.59–1.39)	0.93 (0.59–3.11)	1.02 (0.71–1.46)	1.11 (0.76–1.62)
Age group				
18–29	n/a	1.55 (0.77–3.11)	n/a	0.80 (0.45–1.43)
30–64	n/a	1.21 (0.70–2.11)	n/a	0.65 (0.43–0.99)
65+	Reference		Reference	
Toronto resident	1.62 (1.03–2.53)	1.91 (1.18–3.10)	1.01 (0.66–1.54)	
Non-white ethnicity	1.56 (0.79–3.10)		0.87 (0.41–1.83)	
Canadian-born	0.76 (0.45–1.29)		0.77 (0.49–1.19)	
Secondary level/technical education only	0.54 (0.27–1.06)		0.95 (0.59–1.54)	
Experienced ‘flu-like symptoms’ since April 1, 2009	2.28 (1.45–3.57)		1.90 (1.31–2.77)	
Experienced fever and cough since April 1, 2009	2.69 (1.69–4.29)	2.52 (1.54–4.06)	1.99 (1.30–3.05)	1.99 (1.29–3.07)
Received 08/09 Seasonal flu vaccine	1.81 (1.15–2.86)	1.68 (1.04–2.70)	1.48 (1.01–2.18)	
Received 07/08 seasonal flu vaccine	1.67 (1.02–2.75)		1.17 (0.77–1.77)	
Received 06/07 seasonal flu vaccine	1.48 (0.88–2.48)		1.01 (0.65–1.55)	
Flu vaccine doses				
Three	2.30 (1.20–4.42)		1.33 (0.79–2.23)	
Two	1.53(0.71–3.29)		1.10 (0.59–2.06)	
One	2.02 (0.88–4.63)		1.23 (0.59–2.57)	
None	Reference		Reference	
Previously tested for pH1N1/09	2.65 (0.95–7.38)		1.20 (0.40–3.61)	
Chronic medical condition	1.29 (0.82–2.02)		1.35 (0.92–1.97)	
Currently pregnant	1.37 (0.38–5.00)		0.36 (0.05–2.81)	
Health care worker	1.33 (0.77–2.29)		0.79 (0.45–1.36)	
Lives in a household of 4+ individuals	0.98 (0.59–1.61)		0.83 (0.52–1.33)	
Lives with school-aged children	1.23 (0.75–2.02)		1.08 (0.68–1.72)	
Visited a school or child care centre since April 1, 2009	1.14 (0.75–1.74)		1.23 (0.85–1.78)	
Attended a large public gathering since April 1, 2009	0.85 (0.55–1.33)		0.63 (0.43–0.91)	
Attended a large family gathering since April 1, 2009	1.60 (0.97–2.63)	1.76 (1.03–3.01)	0.97 (0.66–1.42)	
Transit				
Everyday	1.26 (0.65–2.46)		1.06 (0.57–1.95)	
More than once a week	1.42 (0.77–2.62)		0.94 (0.52–1.70)	
Once a week	0.82 (0.43–1.59)		0.69 (0.39–1.21)	
Never	Reference		Reference	
Hand washing				
7 or more times/day	0.92 (0.37–2.28)		0.94 (0.42–2.08)	
3 to 6 times/day	0.79 (0.32–1.97)		0.86 (0.39–1.94)	
0 to 2 times/day	Reference		Reference	

*Aside from age and sex all variables in multivariate model are significant at p<0.05.

Among the 385 seronegative individuals that were followed in the cohort study, 270 received the pH1N1/09 vaccine (table 5). Based on HAI results, among the 95 participants who did not receive the vaccine, 13/95 (13.7%) were seropositive at the end of the 2009/10 influenza season. Forty-nine percent of individuals that received the vaccine were classified as seropositive; however, GMTs were significantly higher among vaccinated participants. Amongst the vaccinated, females (p = 0.0213) and adults under 65 years of age (p = 0.0066) were significantly more likely to remain seropositive at follow up.

**Table 5:** Number and proportion of seropositive participants by HAI and microneutralization and GMT pre and 6–8 months post-vaccine measured by HAI assay among seronegative cohort study participants followed up at the end of the influenza season in April–May 2010.

Vaccine		Total	No. (%) pH1N1/09 seropositive HAI		No. (%) pH1N1/09 seropositive Microneutralization	GMT pre-vaccine (95% CI)	GMT 6–8 month post-vaccine (95% CI)
Received pH1N1/09 vaccine	No	95	13	(13.7)	10 (10.1)	8.5 (5.1–12.0)	10.8 (6.3–15.3)
	Yes	270	133	(49.3)	120 (44.3)	8.3 (5.0–11.7)	30.1 (24.2–35.9)
	Total*	385	154	(40.0)	134 (34.8)	8.4 (5.0–11.7)	23.4 (17.4–29.5)

*pH1N1/09 vaccine status was unknown for 20 participants.

Using age-specific seropositivity rates as well as population estimates in Ontario and baseline seroprevalence in the population, the total outbreak size is estimated between 2.4 and 3.9 million, or 18–30% of the population, implying that routine laboratory surveillance detected approximately 1 in 350 cases of infection Ontario. It is estimated that the infection rate in the second wave was 2.0–3.2 times the size of the first wave. (details provided in appendix S3).

## Discussion

Our results draw a near-complete picture of the evolution of seropositivity to pandemic H1N1from before the onset to the end of the 2009/10 influenza seasons in a large Canadian province, using a variety of study populations and prospectively collected epidemiologic data. Our estimates of infection using the serological data indicate that approximately 24% of the population were infected with pH1N1, which is higher than Hong Kong [10], Australia [11] and New Zealand [12].

Our findings are consistent with laboratory surveillance such that the highest rates of infection were noted in younger age groups, particularly school-aged children [3], [12]–[14]. Consistent with other seroprevalence studies and a systematic review [15], we observed a significant increase from baseline seroprevalence levels among children; however no increase was observed in adults 60 years of age and older [12]. Several seroprevalence studies have demonstrated higher levels of immunity in older age groups [5], [12], [16]–[22]. The higher seroprevalence in the older age groups, particularly those 80 years of age and older, likely represents cross-reactivity due to pre-existing antibodies to previously circulating influenza viruses as sequence comparisons have shown that the hemagglutination gene of pH1N1/09 virus is closely related to 1918 and 1976 viruses [19].

Seroprevalence levels among our preventable disease and prenatal screening populations were similar to two other smaller seroprevalence studies conducted in Canada; however there were important differences [5], [23]. We observed differences in HAI sensitivity compared to MN assay between the first and second waves that were not found in the study from British Columbia [5]. We speculate that the apparent increased sensitivity of HAI in the second wave is consistent with the hypothesis that antibodies detected by MN peaks higher but wanes faster than HAI. The differences may also be due to the fact that two assays target different components of the virus hemagglutinin thus represent different immunological markers. In addition, the risk factor analysis differed between the assays, but this cannot be compared with published seroprevalence studies in Canada that have not included epidemiologic information. We did not observe any association between seroprevalence and either public transit or hand washing found in other settings [24], [25]. This could be due to the recall bias or error that may occur with the self-reporting of this variable.

We estimated of the second wave to be approximately 2.6-fold larger than the first, which is considerably lower than the reported five-fold higher rate in hospitalizations in wave 2 versus wave 1 in Canada [26]. As we measured all infections, compared to only those that were symptomatic enough to warrant hospitalization, this difference would be expected if either the average age at infection or testing of hospitalized patients increased in wave 2.

Study participants who reported receiving seasonal influenza vaccine in 2008/09 were significantly more likely to be seropositive by HAI after adjusting for other factors. Cross-reactivity of vaccine induced antibodies with pH1N1/09 antigen in the HAI assay is an unlikely explanation because the effect size was similar using MN, albeit non-significant. Previous studies have shown increased pH1N1/09 antibody titres among adults who received seasonal influenza vaccinations [20], [21], [27], [28] and four studies conducted in Canada have identified an association between individuals who received the 2008/09 influenza vaccine and becoming infected with medically attended pH1N1/09 [29]. Further examination of the affinity and origin of antibodies detected in previously vaccinated individuals who acquired pH1N1/09 is needed to better understand any potential biological mechanisms.

Ontario distributed Arepanrix™ H1N1 vaccine, an adjuvanted influenza vaccine made by GlaxoSmithKline (GSK). Vaccine coverage within our seronegative cohort study population was 70%, as compared to 33% reported in the general population [30]. This discrepancy may be a result of prior knowledge of susceptibility among participants. Among those who received the pH1N1/09 vaccine, 49% were seropositive by HAI at the end of the influenza season, approximately 6 to 8 months after the vaccine was made available, although GMT levels were significantly higher among the vaccinated group. This was lower than expected from immunogenicity studies carried out by GSK that demonstrated seroconversion rates of 92% and 80% at 42 day follow-up also by HAI [31]. In our study, failure to have detectable antibodies 6–8 months post-vaccination as defined by the 1∶40 cut-off as associated with increasing age, but it is unlikely the findings can solely be explained by our study population being less healthy or older, or by the longer time to follow up. HAI and MN are only proxies for protective immunity; the clinical significance cannot be explored by this methodology. Our findings support the decision made to include the pandemic strain in the 2010/11 seasonal vaccine and recommend it for those who had previously received monovalent vaccine.

Several limitations should be considered when interpreting the results of this study. The residual serum specimens were obtained from predominantly female individuals who were more likely to live in the Toronto region. Some of the selection biases in the overall cohort are likely the result of our web-based methods for recruitment and data collection. Individuals with limited computer skills or access to the internet and individuals who were not fluent in English would have been less likely to participate. The high proportion of health care workers included in the cohort may be due to an increased awareness of our relatively new organization compared to the rest of the population. In addition, our blood collection sites were primarily located in urban centres limiting participants from rural and more isolated Northern communities. Furthermore, we chose the cut-off of 1∶40 for our assays in order to ensure comparability with other studies; different cut-offs would have altered our findings. In a study of the sensitivity and specificity of pH1N1 tests, we found that HI may not be as sensitive as MN even though HI is the commonly accepted method for the detection of antibodies [32]. These differential test performances have implications on the interpretation of serological results for pH1N1. An international evidence-based consensus on the use and interpretation of serology in relation to assessing both immunity and as evidence of recent or past infection would be helpful in this regard. In addition to investigating humoral immunity, it is also of utmost importance to investigate cell-based immunity to explore the cross-reactive memory T cell responses especially considering that antibodies wane over time and some individuals may not produce antibodies [33].

There are various approaches to serological testing in the population, including active population recruitment and passive testing of blood specimens, both of which have for potential selection biases. The advantage of active recruitment is the ability to collect epidemiological information, which can reveal bias and determine risk factors and patterns of serostatus in the population that is not possible through passive data collection. While the selection bias of residual specimens, such as those for prenatal and preventable disease screening, cannot be measured, it may differ from those actively recruited and may not be associated with the outcome. We adopted multiple approaches in order to be more representative of the population. We found synthesizing data from both sources over time extremely useful to represent a more complete picture of the pandemic. Methods to reduce selection bias in serosurveys and the establishment of biorepositories are important future considerations.

This study synthesizes a range of different sources of serological data with prospectively collected epidemiologic information. The collection of data across different time points allows us to observe the evolution of the pandemic, both prior to and after the vaccination program. The results of this seroprevalence study were important because they allowed decision makers to use estimates of susceptibility in the local population to plan resources and support public health action, including vaccination programs, during the second wave and the subsequent influenza season. Before another infectious disease emerges it would be wise to incorporate the need for seroprevalence studies into planning processes and public health emergency plans, establish biorepositories of representative sera as well as the laboratory capacity to test specimens quickly to answer such questions. This experience using multiple assays and population sources will facilitate the introduction of a provincial serosurvey in the event of widespread outbreaks, a future pandemic or other public health emergency. Results are currently being applied to the evaluation of the pandemic response and for pandemic planning.

## Supporting Information

Appendix S1 **Summary of recruitment, sampling methods, and sample size for each study population.**
(DOC)

Appendix S2 **Flow chart of prospective cohort study participants.**
(DOC)

Appendix S3 **Estimation of Outbreak Size using Seroprevalence Data.**
(DOC)
