# Supplementary material for: Seroprevalence of Pandemic Influenza H1N1 in Ontario from January 2009–May 2010
Source: PLoS One. 2011 Nov 14;6(11):e26427. doi: 10.1371/journal.pone.0026427 (PMC3215698; doi:10.1371/journal.pone.0026427)
Supplement: Appendix S1 — Summary of recruitment, sampling methods, and sample size for each study population. (DOC) [file pone.0026427.s001.doc]

| Appendix S1: Summary of recruitment, sampling methods, and sample size for each study population | | | |
| --- | --- | --- | --- |
| **Study Population** | **1) Residual Samples** | | **2) Cohort Study** |
|  | **Preventable Disease Screening** | **Prenatal Screening** |  |
| Source | • Specimens obtained from Toronto, Kingston, Windsor & Hamilton public health laboratories | • Specimens obtained from Toronto public health laboratories | • Prospectively recruited Ontario residents |
| Inclusion criteria | • Samples submitted for mumps, measles & varicella screening | • Samples submitted for prenatal screening | • Adult Ontario residents (18+ years) able to communicate in English & complete web survey |
|  | • Age, sex & FSA entered | • Age, sex & FSA entered | • No travel to Mexico from Jan-April 2009 |
| Specimen collection periods |  |  |  |
| 1. Pre-pandemic | Dec 31, 2008 to Jan 30, 2009 |  |  |
| 2. Early wave 1 pandemic | March 23, 2009 to April 30, 2009 | April 14, 2009 to May 6, 2009 |  |
| 3. Post-wave 1 pandemic | August 13, 2009 to Sept 29, 2009 | Sept 1, 2009 to Sept 18, 2009 | Aug 14, 2009 to Oct 5, 2009 |
| 4. Post-wave 2 pandemic | Dec 30, 2009 to Jan 29, 2010 | Feb 1, 2010 to Feb 26, 2010 |  |
| 5. End of influenza season | April 19, 2009 to May 29, 2010 | April 26, 2010 to May 27, 2010 | April 26, 2010 to July 28, 2010 |
| Targeted sample size | • Toronto: Approximately 60 specimens from each of the following age groups: <18, 18–29, 30–39, 40–49, 50–59, 60–69 and 70+ years for each time period. | • Approximately 25 specimens from each of the following age groups: <18, 18–29, 30–39 and 40–49years for each time period | • Post–wave 1: 600 participants from each the following age groups,18–29, 30–64 and 65+ years |
|  | • Other labs: Approximately 25 from each of the following age groups: <18, 18–39, 40–59 and 60+ years for each laboratory for periods 3*, 4 and 5. |  | • End of influenza season: All seronegative individuals who submitted samples during the post-wave 1 pandemic time period |
| Final sample size |  |  |  |
| 1. Pre-pandemic | 383 | - | - |
| 2. Early wave 1 pandemic | 383 | 105 | - |
| 3. Post-wave 1 pandemic | 505 | 120 | 1024 |
| 4. Post-wave 2 pandemic | 838 | 136 | - |
| 5. End of influenza season | 790 | 115 | 373 |
| Laboratory testing | HAI assays | HAI assays | HAI & MN assays |
| *Residual specimens from the Kingston laboratory site were unavailable for Period 3. | | | |
