# Supplementary material for: Seroprevalence of Pandemic Influenza H1N1 in Ontario from January 2009–May 2010
Source: PLoS One. 2011 Nov 14;6(11):e26427. doi: 10.1371/journal.pone.0026427 (PMC3215698; doi:10.1371/journal.pone.0026427)
Supplement: Appendix S2 — Flow chart of prospective cohort study participants. (DOC) [file pone.0026427.s002.doc]

Appendix S2: Flow chart of prospective cohort study participants

Ontarians registered

N= 1486

**TOTAL post wave 1 analysis**

**1024**

Seronegative individuals from post wave 1

941

**TOTAL end of influenza season**

**385**

Withdrew

53 (3.6%)

Actively Withdrew

38 (4.0%)

Passively withdrew

518 (57.4%)

Completed the online questionnaire

1245 (86.9%)

Had a blood specimen collected for testing

1069 (74.6%)

Removed from post wave 1 analysis due to being tested after Oct. 5 2009

45 (4.2%)
