# Supplementary material for: Seroprevalence of Pandemic Influenza H1N1 in Ontario from January 2009–May 2010
Source: PLoS One. 2011 Nov 14;6(11):e26427. doi: 10.1371/journal.pone.0026427 (PMC3215698; doi:10.1371/journal.pone.0026427)
Supplement: Appendix S3 — Estimation of Outbreak Size using Seroprevalence Data. (DOC) [file pone.0026427.s003.doc]

Appendix S3: Estimation of Outbreak Size using Seroprevalence data

Wave 1

|  | A | B | C | D | E |
| --- | --- | --- | --- | --- | --- |
| Age Group | 2009 Ontario  Population | Pre-pandemic  immunity (%) ^1^ | Total post-wave 1  Immunity (%) ^1^ | pH1N1 infection  post -wave1  (C - B) | Population  Infections  Post-wave 1 (A x D) |
| <18 | 2,729,827 | 2 | 21·6 | 19·6 | 535,046 |
| 18–29 | 2,167,706 | 7.4 | 12·5 | 5·1 | 110,553 |
| 30–64 | 6,383,722 | 4.1 | 11 | 6·9 | 440,477 |
| 65+ | 1,787,927 | 12 | 19 | 7·0 | 125,155 |
| Total | 13,069,182 |  |  |  | 1,211,231 |

^1^ From Table 3

Outbreak size of wave 1 = 1,211,231 (E from table)

Wave 2

|  | F | H | I | J |
| --- | --- | --- | --- | --- |
| Age Group | 2009 Ontario  Population  less immune  (A – E) | End of season  Immunity (%) ^1^ | pH1N1  post -wave 2  less baseline  and wave 1 (%) *  (H - C) | Number pH1N1  immunity post  wave2*  (I x F) |
| <18 | 2,194,781 | 61·7 | 42·1 | 924,003 |
| 18–29 | 2,057,153 | 37·9 | 32·8 | 674,746 |
| 30–64 | 5,943,245 | 36·3 | 29·4 | 1,747,314 |
| 65+ | 1,662,772 | 34·8 | 27·8 | 462,251 |
| Total | 11,857,951 |  |  | 3,808,314 |

^1^ From Table 3

* includes infection and vaccination

Wave 2 estimates

|  | J | N | O |
| --- | --- | --- | --- |
| Age Group | pH1N1immunity post  wave2*  (I x F) | Number immune due to vaccine  Assumption 1:  Percent immune due to vaccine = 50%  (0·50 x J) | Total immune  Less vaccine Assumption 1  (J - N) |
| <18 | 924,003 | 462,002 | 462,002 |
| 18–29 | 674,746 | 337,373 | 337,373 |
| 30–64 | 1,747,314 | 873,657 | 873,657 |
| 65+ | 462,251 | 231,126 | 231,126 |
| Total | 3,808,314 | 1,904,157 | 1,904,157 |

Outbreak size of wave 2 = 1,904,157 (O)

Total Outbreak size: W1 + W2 = 1,211,231 +1,904,157 = 3,115,388

Sensitivity analysis on N assumption (range from 30% – 70%)

|  | J | P | Q |
| --- | --- | --- | --- |
| Age Group | pH1N1  immunity post  wave2*  (I x F) | Total immune less vaccine immunity  Assumption 2:  Percent immune due to vaccine = 30%  (0·70 x J) | Total immune less vaccine immunity  Assumption 2:  Percent immune due to vaccine = 30%  (0·30 x J) |
| <18 | 1,149,257 | 646,802 | 277,201 |
| 18–29 | 711,008 | 472,322 | 202,424 |
| 30–64 | 1,876,814 | 1,223,120 | 524,194 |
| 65+ | 497,044 | 323,576 | 138,675 |
| Total | 4,234,123 | 2,665,820 | 1,142,494 |

Range of Outbreak Size:

Total Outbreak size: W1 + W2 = E + Q 1,211,231 + 1,142,494= 2,353,725 (18% of population)

Total Outbreak size: W1 + W2 = E + P = 1,211,231 + 2,665,820= 3,877,051 (30% of population)
